# Population-based screening strategies for biliary atresia in the newborn: A systematic review and meta-analysis

**DOI:** 10.1371/journal.pone.0307837

**Published:** 2024-08-28

**Authors:** Srirupa Hari Gopal, Rema Zebda, Arvind Mohan, Kristin Borovsky, Yemisi Takwoingi, Katie Scandrett, Mohan Pammi

**Affiliations:** 1 Division of Neonatal-Perinatal Medicine, Baylor College of Medicine & Texas Children’s Hospital, Houston, Texas, United States of America; 2 University of Pennsylvania, Philadelphia, PA, United States of America; 3 Division of Pediatric Gastroenterology, Baylor College of Medicine & Texas Children’s Hospital, Houston, Texas, United States of America; 4 Institute of Applied Health Research, College of Medical and Dental Sciences, University of Birmingham, Birmingham, United Kingdom; Children’s Hospital of Eastern Ontario (CHEO), University of Ontario, CANADA

## Abstract

**Background:**

Newborn screening for biliary atresia (BA) may facilitate earlier diagnosis and intervention for improved clinical outcomes.

**Methods:**

We systematically reviewed the accuracy of population-based screening strategies for BA in the newborn using PRISMA-DTA guidelines. We included cohort or cross-sectional studies. The screening (index) tests included stool color card (SCC) and direct/conjugated bilirubin (DB/CB) and the reference standard was intraoperative cholangiogram. Meta-analysis was performed using random-effects logistic regression models.

**Results:**

We included 15 studies (1,816,722 participants) that assessed 5 different population-based screening strategies. QUADAS-2 assessment revealed high risk of bias for patient selection in one study and uncertain risks for reference standard in multiple studies. High certainty evidence suggests that DB/CB assessed after birth had a summary sensitivity of 100% (95% CI 100,100) and specificity of 98.8% (98.8,98.9) (5 studies, 662141 participants). Moderate certainty evidence suggests that SCC screening at a month of age had summary sensitivity of 79.6% (95% CI 70.6, 86.4) and specificity of 99.9% (95% CI 99.9, 99.9) (7 studies, 996262 participants).

**Conclusions:**

DB/CB in the first few days of life has the best diagnostic accuracy for population screening for biliary atresia in the newborn. Future research should focus on cost-effectiveness and combinations of screening strategies.

## Introduction

Biliary atresia (BA) is the leading indication for pediatric liver transplant, affecting between 1 in 8,000 and 1 in 18,000 infants [1]. BA leads to fibrosis of the bile ducts, resulting in cholestasis, and can progress to complications such as portal hypertension, growth failure, cirrhosis and ultimately death due to end-stage liver disease [1]. Variability exists in the incidence of this disorder based on geographical region and ethnicity, with higher incidence in the Asia and Pacific regions [2, 3]. The incidence varies from one to five per 10,000 live births in Taiwan and Japan, [2–6] to one per 15–20,000 live births in UK, Ireland, France and Canada [2, 3, 7–9] and approximately between 6.5 to 7.5 per 100,000 live births in the US. The rapid course of biliary atresia can be slowed with the Kasai portoenterostomy (KPE), a surgical intervention performed to establish bile flow by removing atretic bile ducts and creating a liver-intestine anastomosis using a Roux-en-Y loop anastomosis [1, 10].

The timing of diagnosis and the KPE procedure impacts the prognosis of patients with biliary BA. In a study by Serinet et al, it was reported that KPE performed in the first 46 days of life was associated with 65.5% survival rates with native liver at two years of age [11]. Recent studies have also found that treatment by 30 days of life delayed or prevented the need for a liver transplant [8, 12]. However, BA is known to have a latent preclinical phase, which may impact the timing of diagnosis and subsequently delay management and worsen outcomes. In the US, the average age at referral was 53 days and average age at KPE was 61 days [13].

The demand for the implementation of newborn screening strategies for the early diagnosis and management of BA has grown [9, 14, 15]. Several population-based screening strategies have been implemented for early detection and management of this disease. Screening strategies include stool color card (SCC), serum direct or conjugated bilirubin (DB/CB) measurements, urine sulfated bile acids (USBA), serum bile acids and serum free carnitine. Current evidence regarding the accuracy of population-based screening strategies for BA has not been adequately summarized.

### Aims of current review

1. To perform a systematic review and meta-analysis of the diagnostic accuracy of population-based screening strategies for early diagnosis of biliary atresia in the newborn.
2. To compare the accuracy of the different screening strategies for BA.

## Methods

Our protocol was registered with PROSPERO (registration number CRD42022346461)

(Available from: https://www.crd.york.ac.uk/prospero/display_record.php?ID=CRD42022346461)

### Search strategy

We searched the following electronic databases in July 1, 2022, and updated in Feb 2023 (**S1 File)**:

1. MEDLINE, Cumulative Index to Nursing and Allied Health Literature (CINAHL) through EBSCOhost, Embase, and the Cochrane Central Register of Controlled Trials (CENTRAL) in the Cochrane Library.
2. Abstracts of conferences: Pediatric Academic Societies (American Pediatric Society, Society for Pediatric Research, and European Society for Pediatric Research) from 1990 in the journal Pediatric Research, and at https://www.pas-meeting.org/past-abstracts/online from the year 2017 to 2022.
3. Ongoing trials at the following web sites: www.ClinicalTrials.gov, www.controlled-trials.com and https://www.who.int/clinical-trials-registry-platform.
4. PubMed’s related citations feature was used to identify relevant articles. Additional searches were made from articles cited by the included studies and by contacting experts in the field.

### Inclusion criteria

#### Types of studies

We included prospective or retrospective, cohort or cross-sectional studies that evaluated population-based screening for diagnosis of BA in the newborn. We excluded studies that assessed the diagnostic accuracy of the test using only positive samples or healthy controls (case-control study design), and studies not in the clinical context of population screening for BA.

#### Types of participants

Newborn aged six weeks or younger.

#### Index tests

SCC, serum DB/CB, Urine sulfated bile acids (USBA), serum bile acids and free carnitine using any kit and method of assay.

#### Target condition and reference standard

BA diagnosed by intraoperative cholangiogram and/or tissue pathology following Kasai procedure.

### Exclusion criteria

Studies that were not population-based and those that did not address screening were excluded.

## Data collection and analyses

### Selection of studies

Two researchers (MP and AM) independently screened the titles and abstracts and selected articles for full text review. MP and AM retrieved the selected full texts, and independently evaluated them for eligibility, as detailed in our inclusion and exclusion criteria. The articles were further independently reviewed by three researchers (SHG, RZ and KB) for eligibility for the index test, reference standard, patient population and the review question. We resolved any disagreements through discussion and consensus.

### Data extraction and management

Three researchers (SHG, RZ and KB) independently extracted data from each of the included studies (**S2 File).** The extracted data included author, year of publication, study design, inclusion criteria, exclusion criteria, reference standard and performance of the test, index test and performance of the test, blinding information, information about Quality Assessment of Diagnostic-Accuracy Studies (QUADAS-2) [16] and 2x2 table data (number of true positives, false positives, false negatives, true negatives). Sensitivities and specificities were calculated from the 2x2 tables. For the studies that only reported sensitivity and specificity, a reverse calculation was done to obtain the 2x2 table. We also sought additional information about the study design or data, if needed, from the authors of the included studies via email. Any disagreements were resolved by the senior author (MP).

### Assessment of methodological quality

The methodological quality of each study was assessed using the QUADAS-2 tool (as recommended by Cochrane; https://methods.cochrane.org/sdt/) by review authors SHG, RZ and KB [16]. The QUADAS-2 tool was tailored to the review question by modifying specific signaling questions. We assessed four domains for risk of bias: patient selection, index test, reference test, and flow and timing. We assessed applicability concerns in the first three domains (patient selection, index test, and reference test). For each domain, we answered questions with a Yes/No/Unclear answer, and assessed risk of bias as Low/High/Unclear (**S3 File**). Quality assessment regarding eligibility of the studies was performed by two independent authors (SHG and RZ).

### Assessment of the certainty of the evidence

We assessed certainty of evidence used the GRADE approach, as outlined in the GRADE Handbook [17–19]. Two review authors independently assessed the certainty of evidence for SCC and DB/CB population-based newborn screening strategies. We used the GRADEpro Guideline Development Tool to create ‘Summary of findings’ (SoF) tables to report the certainty of the evidence (GRADEpro Guideline Development Tool [Software]. McMaster University and Evidence Prime, 2022. Available from gradepro.org).

### Statistical analysis and data synthesis

The extracted data was entered into Review Manager (RevMan Version 5.4, The Cochrane Collaboration, 2020). A descriptive analysis of the included studies was performed by generating forest plots to visually display individual estimates of sensitivity and specificity. Previous literature suggests that the specificity of screening tests for BA is high [20–34]. Thus, the correlation between sensitivity and specificity across studies is unlikely to be high [35, 36]. Therefore, random-effects logistic regression models were fitted separately for sensitivity and specificity using the ‘meqrlogit’ command in Stata 17.0. If the random-effects logistic regression analyses failed to converge, estimates and confidence intervals were computed by summing the counts of true positive, false positive, false negative and true negative across the 2x2 tables. The 95% confidence intervals was calculated using the Wald method [37].

## Results

### Search results and study selection

**Fig 1:**
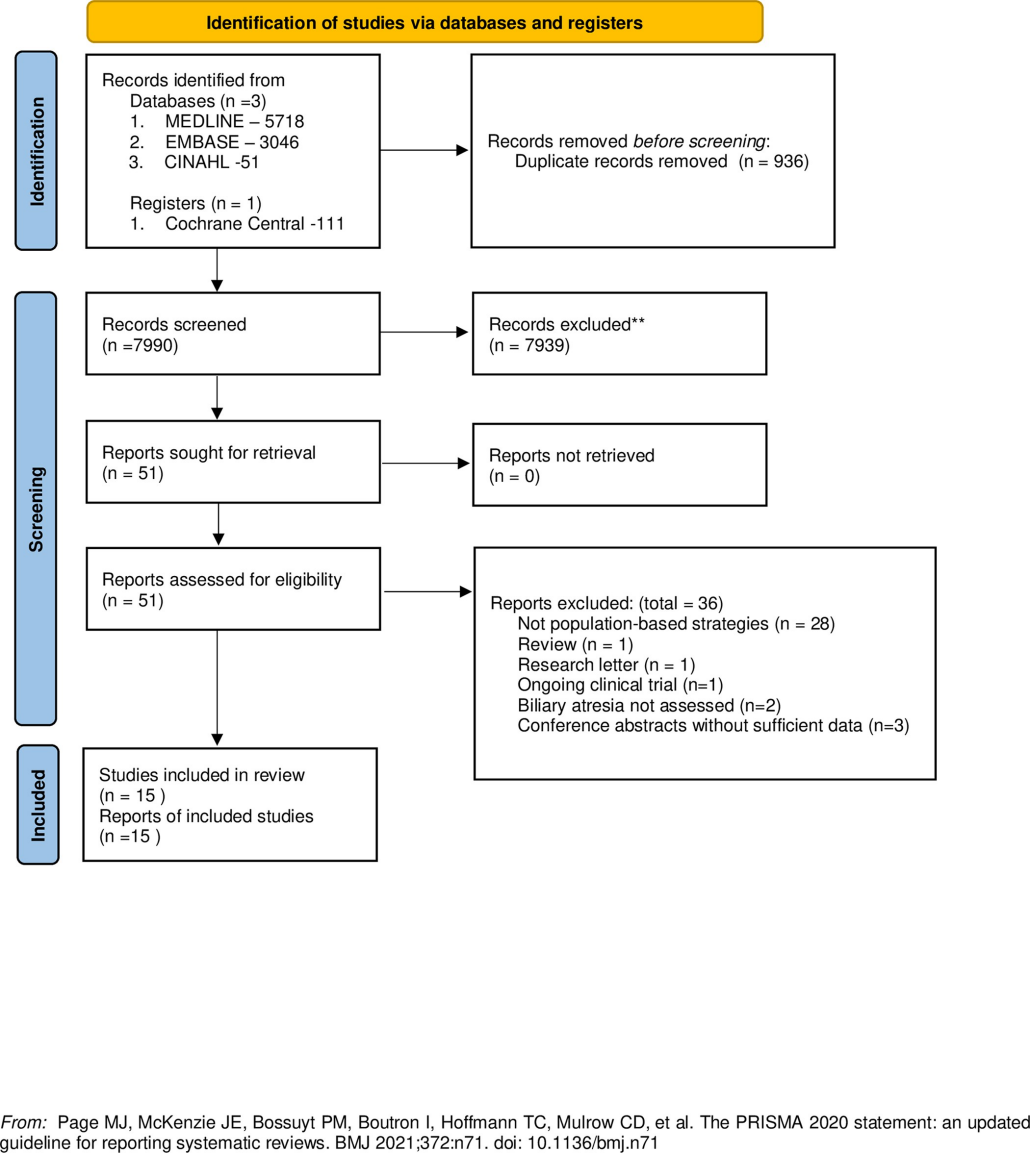
PRISMA flow diagram. Flow diagram describing the study selection process, included and excluded studies for this systematic review and meta-analysis.

We identified 8,926 records through database searches. The inclusion process is detailed in the PRISMA flow diagram (**Fig 1**). We included 15 studies 20–34 of which 7 studies assessed SCC, 5 assessed screening with DB/CB, 1 each assessed USBA, serum bile acids and free carnitine (**Table 1**). The included studies were primarily conducted in Asia (4 from Japan, 3 from China, and 2 from Taiwan) where the reported incidence of BA is known to be higher than in Western nations.4–6 Outside Asia, studies were performed in the USA (4 studies), Canada (2 studies) and the UK (1 study). Excluded studies and reasons for exclusion are described in **S4 File**.

**Table 1:** Characteristics of included studies.

Author, year, country	Sample Size	Study Design	Criteria	Index Test	Reference Standard
Chen, 2006, Taiwan [20]	78,184	Prospective	All newborns from a total of 49 hospitals and clinics covering the northern, central, southern, and eastern parts of Taiwan from March 2002-Dec 2003.	Stool Color Card	Not specified
Gong, 2020, China [21]	233,000	Retrospective	All newborns from a total of 49 hospitals and clinics covering the northern, central, southern, and eastern parts of Taiwan from March 2002-Dec 2003.	Free Carnitine	Liver biopsy /Intraoperative cholangiogram
Gu, 2015, Japan [22]	313,230	Retrospective	All infants born to mothers living in Tochigi prefecture from August 1994 to March 2011.	Stool Color Card	Intraoperative cholangiogram or laparotomy
Gu, 2020, China and Japan [23]	65,039	Retrospective	SCC data were collected from December 2013 to October 2014 in ChaoYang district of Beijing; from April 2012 to July 2015 in Sapporo during routine works in Sapporo City Institute of Public Health.	Stool Color Card	Not specified
Guthery, 2019, USA [24]	25,120	Retrospective	Newborns born between January 2005 to March 2019, including patients who had fractionated bilirubin obtained and length of stay less than 96 hrs.	Direct and Conjugated Bilirubin	Not specified
Harpavat, 2016, USA [25]	11,636	Prospective	Infants born in 4 Houston hospitals in a 4-month period.	Direct and Conjugated Bilirubin	Cholangiogram
Harpavat, 2020, USA [26]	124,385	Prospective	Infants born at 14 Texas hospitals between January 2015 and January 2018.	Direct and Conjugated Bilirubin	Intraoperative cholangiogram or liver biopsy
Hsiao, 2008, Taiwan [27]	422,273	Prospective	Infants born between January 2004 and December 2005 in Taiwan.	Stool Color Card	Intraoperative cholangiogram or liver biopsy
Kastenburg, 2023, USA [28]	252,892	Retrospective	All newborn infants born between 2005–2013 who had a fractionated bilirubin	Conjugated and unconjugated bilirubin	Intraoperative cholangiogram and/or histological features of BA
Kong, 2016, China [29]	29,799	Prospective	Infants born December 2013 to October 2014 in 25 maternal facilities located in the Chaoyang district of Beijing.	Stool Color Card	Laparoscopic surgery
Matsui, 1993, Japan [30]	104,785	Prospective	Infants born between April 1987 and March 1992, in Tochigi Prefecture, Japan.	Total 3-alpha-OH Bile Acid	Intraoperative cholangiogram or laparotomy
Powell, 2003, United Kingdom [31]	30,079	Prospective	All babies under 28 days old having routine neonatal screening for PKU and CHT in the Birmingham health district between August 1995 and July 1997.	Conjugated and Unconjugated Bilirubin	Not specified
Suzuki, 2011, Japan [32]	1,855	Prospective	Infants born at Juntendo University, Nagasaki University, and their affiliated hospitals.	Stool Color Card	Intraoperative cholangiogram
Schreiber, 2014, Canada [33]	6,187	Prospective	Healthy newborns discharged within 2 weeks of birth from BC Women’s Hospital, Vancouver between December 2010 to December 2011.	Urinary Sulfated Bile Acid	Not specified
Woolfson, 2018, Canada [34]	87,583	Prospective	All live neonates born in British Columbia between April 2014 and March 2016 discharged from maternity ward.	Stool Color Card	Intraoperative cholangiogram

### Methodological quality of included studies

**Fig 2:**
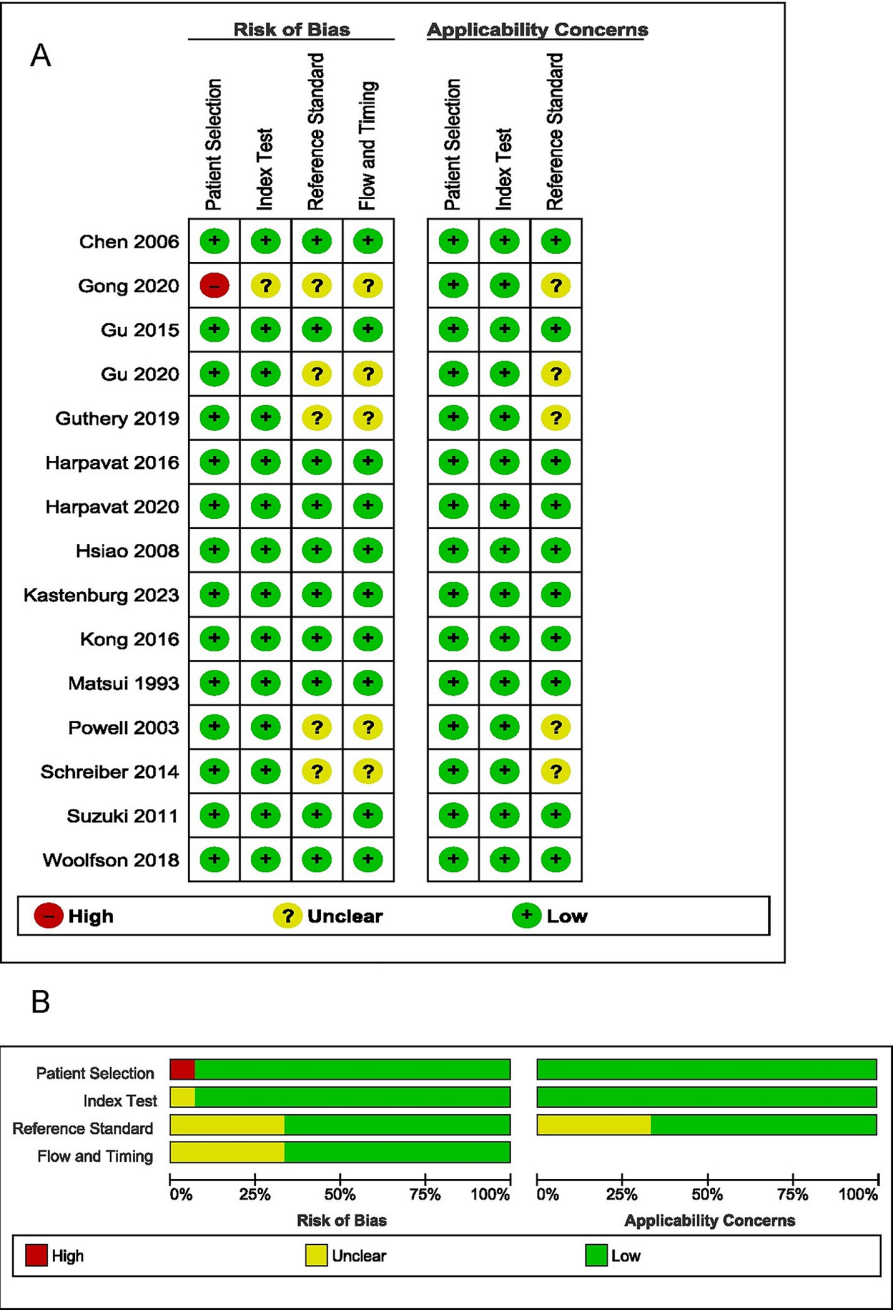
QUADAS methodological assessment of included studies. **2A** risk of bias and applicability concerns summary: review authors’ judgements about each domain for the included study and **2B** risk of bias and applicability concerns graph: review authors’ judgements about each domain presented as percentages across included studies.

Quality assessment using the QUADAS-2 tool revealed deficiencies in the following domains (**Fig 2A and 2B and S3 File**):

1. **Patient selection.** Most included studies were conducted either retrospectively or prospectively at a population level, ideal for assessing accuracy of a screening test. However, one paper, [21] retrospectively identified patients with BA, introducing a high risk of bias via patient selection. Inclusion and exclusion criteria varied between studies, though many excluded infants that were not discharged from a newborn nursery (i.e., those born prematurely and/or with another serious medical condition), a potential source of patient selection bias.
2. **Index test.** Most studies had a predefined threshold for positive screen for BA. Studies that used the SCC, had predefined criteria for a positive test and studies that assessed DB/CB had predefined cut-off thresholds for a positive screen.
3. **Reference standard.** All studies that reported a reference standard utilized an intraoperative cholangiogram for final diagnosis of BA. However, not all studies explicitly stated how BA diagnosis was confirmed.
4. **Flow and timing.** There were significant variability in timing between when screening tests were conducted, ranging from at birth in the DB/CB studies to within the first few months of life with SCC screening. A majority of the studies did not report the time interval between when a screening test (index test) was conducted and when confirmation of BA was diagnosed with an intraoperative cholangiogram (reference standard).

### Meta-analysis for summary estimates of sensitivity and specificities

**Fig 3:**
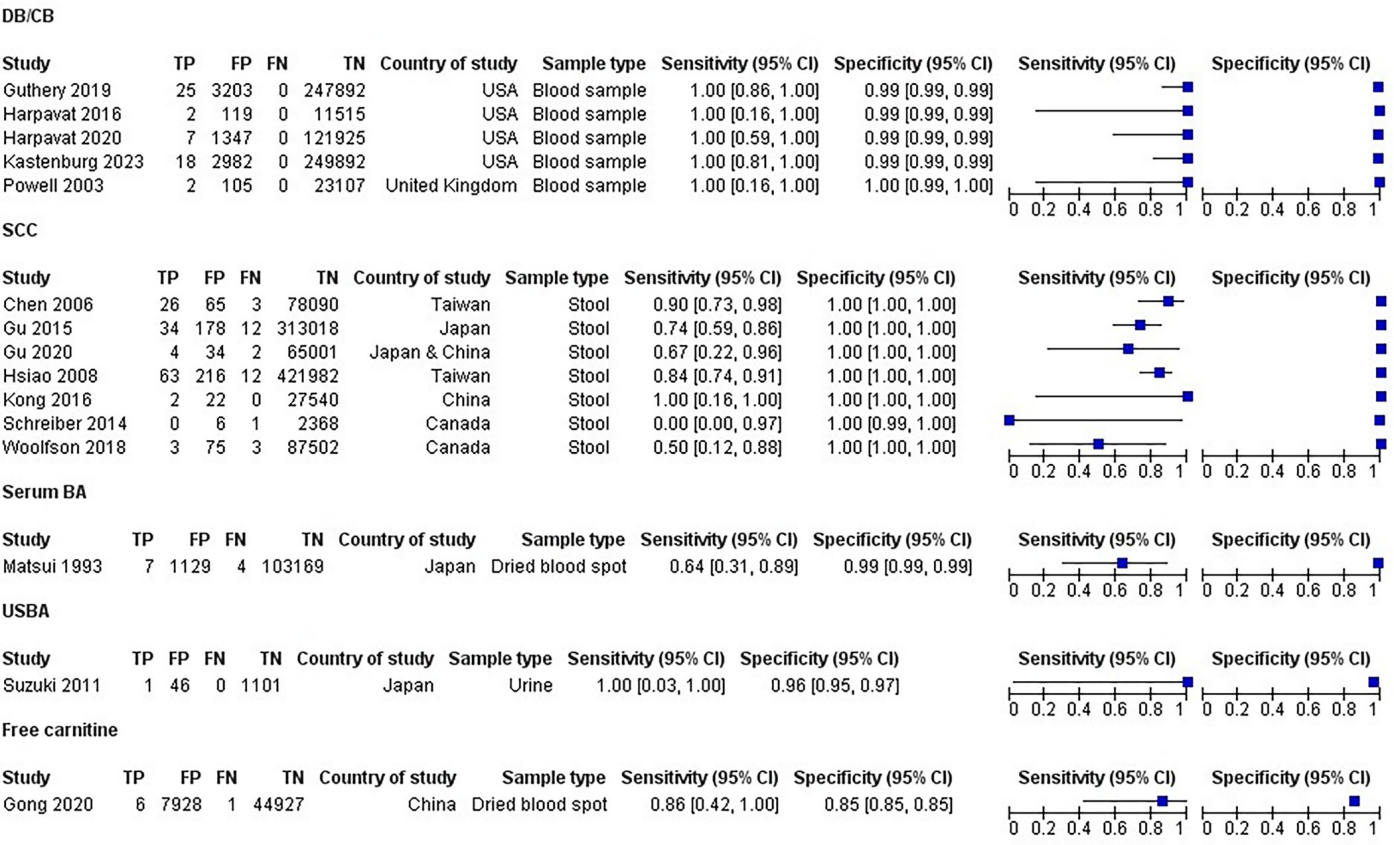
Forest plot of the included studies for screening biliary atresia in the newborn. Sensitivity and specificity for the individual included studies for the five biliary atresia screening are shown in separate left and right panels. Forest plots allow for visual assessment of variability in sensitivity and specificity among studies.

SCC screening had sensitivity ranging from 50% to 100% and specificity of 100% in the 7 included studies. DB/CB had a sensitivity of 1.00 for detecting BA in all 5 included studies and specificity ranged from 99% to 100%. **Fig 3** shows the forest plot displaying individual estimates of sensitivity and specificity from each included study.

Estimated summary sensitivity by meta-analysis (**S1 Table)** for studies evaluating DB/CB was 100% (95% CI 100, 100) and specificity was 98.8% (95% CI, 98.8, 98.9). Estimated summary sensitivity for studies evaluating SCC was 79.6% (95% CI, 70.6,86.4) and specificity was 99.9% (95% CI, 99.9, 99.9). The following screening tests were only assessed by one study each and reported the following sensitivities and specificities: USBA in one included study [32], had a sensitivity of 100% (95% CI, 0.03–1.0) and a specificity of 96% (95%CI, 0.95–0.97); serum bile acids had a sensitivity of 64% (95% CI, 0.31–0.89) and a specificity of 99% (95% CI, 0.99–0.99) (1 study^30^); free carnitine had a sensitivity of 86% (0.42–1.00) and a specificity of 85% (0.85–0.85) (1 study [21]).

### Assessment for certainty of evidence

GRADE scale [17–19] showed high certainty of evidence for DB/CB screening and a moderate certainty of evidence for SCC screening (downgraded for inconsistency) for BA in newborn (**S5 File**). USBA, serum bile acids and free carnitine screening strategies were graded as very low certainty evidence, downgraded due to suspected publication bias, indirectness and imprecision.

## Discussion

We reviewed and summarized the diagnostic accuracy of different population-based screening strategies for the diagnosis of BA in the newborn using Cochrane methodology. Fifteen studies assessed 5 different population-based screening strategies in 15 studies (1,816,722) participants were included. Risk of bias was assessed using the QUADAS-2 assessment tool and most included studies scored low or unclear for risk of bias and applicability concerns with the exception of high risk of bias for patient selection in one study,21 which identified patients with BA retrospectively introducing a high risk of bias via patient selection. Some studies did not explicitly state the reference standard used although implied and hence scored as uncertain risks for reference standard [21, 23, 24, 31, 33]. Using GRADE criteria, the certainty of evidence was high for screening with DB/CB and moderate for SCC (downgraded certainty for imprecision and inconsistency) and very low certainty for USBA, serum bile acids and free carnitine measurements.

DB/CB screening has high sensitivity and specificity for detecting BA, when incorporated in newborn screening after birth and is also easily implemented, either at the birthing institution or a pediatrician’s office. SCC screening was noted to have high specificity but moderate sensitivity. Both DB/CB and SCC are minimally invasive with SCC screening being completely painless, and DB/CB requiring one to two heel sticks, which are typically already performed for a newborn screen and/or 24 hour of life bilirubin testing. USBA, serum bile acids, and free carnitine tests were assessed in only one population-based study each (which were assessed as very low certainty) but these tests are not routinely performed in pediatric offices or hospitals, which would make them challenging and likely expensive to implement across a wide population.

In the included studies of the review, variability existed in the timing of screening tests among screening strategies. DB/CB, serum bile acids, and free carnitine are all screening methods performed at or near time of birth. In comparison, USBA were collected within the first 10–40 days of life and SCC screening was performed over the first few weeks of life. The studies that utilized SCC screening primarily used the Taiwan National Stool Color Card, which includes 7 images of stool (images 1–3 represent abnormal stool color and 4–7 represent normal stool color) [20, 23, 29]. Other included studies assessing SCC screening utilized an International Stool Color Card [27, 33, 34] All but one of the included studies that assessed the DB/CB method of screening were similar in their threshold for test positivity (CB >0.2 mg/dL and DB >0.5 mg/DL or 97.5% for the lab) [24–26, 28]. Powell et al used a higher threshold of CB >18 mmol/L and fraction (100x CB/[CB+UB]) >20% [31].

Due to differences in the timing of the DB/CB (around the time of birth) and SCC (screening within the first few weeks of life, likely at the monthly pediatrician visit) tests, a two-stage screening strategy may be feasible. Infants could have DB/CB checked at birth and, if elevated, are further worked up for BA with a pediatric gastroenterologist. However, if the family is lost to follow-up or the initial bilirubin is normal, pediatricians can then do an in-office SCC screen at the two-four week visit to screen for acholic stools, providing another step in the screening process in which a child with BA could still be diagnosed early in the disease process. Detecting BA earlier with high diagnostic accuracy (high sensitivity and specificity) will result in earlier and more functioning KPEs, fewer liver transplants at a young age, and improved clinical outcomes overall.

Multiple studies that assessed screening strategies for BA were excluded from our analysis since they were not performed at a population level (**S4 File**). It is crucial that potential screening tools are assessed at the population level, and not among a smaller cohort of non-representative individuals (e.g. cholestatic infants) since this may lead to spectrum bias, which describes the differing performance of a diagnostic test among different populations. Results from a study where a screening test for BA was performed on known cholestatic infants or retrospectively identified infants with BA would also mean that the results are not generalizable to all infants, which is the population a national screening test would be implemented on.

### Clinical applicability

In a hypothetical cohort of 100,000 newborn infants where DB/CB is used for BA screening (approximate pre-test probability is 0.0067%, 1 in 15,000 infants, derived from published studies), applying the summary sensitivity of 1.00 and summary specificity of 0.98 from this review, no patients with the disease will be missed by DB/CB. There will be 2000 false positives or patients diagnosed with BA. If SCC is used for BA screening in the same hypothetical cohort of 100,000 newborn infants, (pre-test probability is 0.0067%, 1 in 15,000 infants), applying the summary sensitivity of 0.79 and summary specificity of 0.99 from this review, one patient with the disease will be missed by SCC and there will be 1000 false positives or patients diagnosed with BA. Since these are screening tests (not diagnostic tests) for a relatively rare disease but with severe consequences, not missing a case of BA takes priority and the false positives are further investigated by the pediatric gastroenterologist or other healthcare provider.

### Strengths and limitations of the review

This is a methodologically robust systematic review and screening test accuracy meta-analysis to summarize the diagnostic accuracy of different screening methods for biliary atresia at the population level. Our protocol was pre-registered with PROSPERO. Our review includes a recent large population-based study published in early 2023 [28] and was performed according to standard recommendations of the Cochrane DTA methodology group (https://methods.cochrane.org/sdt/). Inclusion in our review was limited to true population-based studies and the QUADAS-2 tool was appropriately utilized for methodological assessment of screening tests [16]. We assessed the certainty of evidence using the GRADE approach to inform clinical practice and research.

We recognize that heterogeneity exists in the included studies due to the timing of screening and differences in the populations screened. Heterogeneity is a well-recognized problem in systematic reviews of screening and/or diagnostic tests [38]. Two of the included studies required infants to be in a newborn nursery for study inclusion, which excluded infants admitted to the NICU [32, 34]. Prematurity may be associated with the occurrence of BA, exclusion of preterm infants in the NICU may have introduced bias and affect applicability [39].

## Conclusions

### Implications for practice

High certainty evidence suggests that DB/CB screening performed in the first few days of life has high diagnostic accuracy for BA in the newborn. Moderate certainty evidence suggests that SCC has high specificity but only moderate sensitivity when performed in the first few weeks of life.

### Implications for research

Further research is warranted to investigate the cost-effectiveness of implementing these screening strategies, alone or in combination. It is also of interest to investigate how early diagnosis due to the suggested screening strategies impacts timing of KPE and the need for liver transplant.

## Supporting information

S1 Checklist PRISMA 2020 checklist. (DOCX)

S1 File Search strategies for biliary atresia and neonatal screening: Detailed description of search strategy employed for the systematic review. (DOCX)

S2 File Data extraction. Description of various items extracted from the included studies for the systematic review.
(DOCX)

S3 File QUADAS-2 assessment of included studies. Detailed description of each study on QUADAS-2 assessment.
(DOCX)

S4 File Table of excluded studies. Details on studies that were excluded from the metanalysis and reasons for exclusion.
(DOCX)

S5 File GRADE certainty of evidence for included studies. Tables showing GRADE certainty of evidence for both direct/conjugated bilirubin and Stool Color Card test for screening of biliary atresia.
(DOCX)

S1 Table Summary sensitivity and specificities of screening strategies for biliary atresia using stool color card (SCC) test and direct/conjugated bilirubin (DB/CB). (DOCX)
