# Supplementary material for: Population-based screening strategies for biliary atresia in the newborn: A systematic review and meta-analysis
Source: PLoS One. 2024 Aug 28;19(8):e0307837. doi: 10.1371/journal.pone.0307837 (PMC11357077; doi:10.1371/journal.pone.0307837)
Supplement: S1 File — (DOCX) [file pone.0307837.s002.docx]

**Supporting Information 1:** Search strategies for biliary atresia and neonatal screening

1. Ovid MEDLINE(R) and Epub Ahead of Print, In-Process, In-Data-Review & Other Non-Indexed Citations and Daily <1946 to June 24, 2022>

# Query Results

1 'direct bilirubin'.mp, 🡪 2,112

2 'conjugated bilirubin'.mp. 🡪 955

3 fractionated bilirubin.mp. 🡪 14

4 Stool color card.mp. 🡪 39

5 SCC.mp. 🡪 23,956

6 Bile acids.mp. or "Bile Acids and Salts"/ 🡪 30,981

7 Taurocholate.mp. or Taurocholic Acid/ 🡪 6,686

8 Screening.mp. 🡪 705,062

9 1 or 2 or 3 or 4 or 5 or 6 or 7 or 8 🡪765,517

10 jaundice.mp. or Jaundice, Neonatal/ or Jaundice/ or Jaundice, Chronic Idiopathic/ or Jaundice, Obstructive/ 🡪 44,884

11 Cholestasis, Intrahepatic/ or cholestasis.mp. or Cholestasis/ or Cholestasis, Extrahepatic/ 34,285

12 biliary atresia.mp. or Biliary Atresia/ 🡪 5,466

13 (Kasai procedure or kasai or portoenterostomy).mp. [mp=title, abstract, original title, name of substance word, subject heading word, floating sub-heading word, keyword heading word, organism supplementary concept word, protocol supplementary concept word, rare disease supplementary concept word, unique identifier, synonyms] 🡪 1,783

14 10 or 11 or 12 or 13 🡪74,669

15 exp Infant, Newborn/ 🡪 655,642

16 Neonatal Nursing/ or Female/ or Neonatology/ or Infant, Newborn/ or Neonatal Screening/ or Neonatal Sepsis/ or Humans/ or Male/ 🡪22,768,128

17 newborn*.mp. 🡪815,045

18 15 or 16 or 17 🡪22,844,971

19 9 and 14 and 18 🡪 5,718

2. **EMBASE**

Id.  Query                                                  Publications-
#16. ((direct AND ('bilirubin'/exp OR bilirubin)) OR         3,046 
     (conjugated AND ('bilirubin'/exp OR bilirubin))
     OR (fractionated AND ('bilirubin'/exp OR
     bilirubin)) OR 'stool color card' OR scc OR 'bile
     acids' OR taurocholate OR screening) AND
     (jaundice OR (biliary AND atresia) OR
     'cholestasis' OR 'portoenterostomy') AND
     'newborn'
#15. 'newborn'                                               793,426 
#14. jaundice OR (biliary AND atresia) OR                   145,879 
     'cholestasis' OR 'portoenterostomy'
#13. 'portoenterostomy'                                       2,331 
#12. 'cholestasis'                                           61,241 
#11. biliary AND atresia                                      8,553  #10. jaundice                                                93,328 
#9.  (direct AND ('bilirubin'/exp OR bilirubin)) OR       1,335,499 
     (conjugated AND ('bilirubin'/exp OR bilirubin))
     OR (fractionated AND ('bilirubin'/exp OR
     bilirubin)) OR 'stool color card' OR scc OR 'bile
     acids' OR taurocholate OR screening
#8.  screening                                            1,256,216 
#7.  taurocholate                                             6,752  25
#6.  'bile acids'                                            23,426 
#5.  scc                                                     40,414 
#4.  'stool color card'                                          63 
#3.  fractionated AND ('bilirubin'/exp OR bilirubin)            230 
#2.  conjugated AND ('bilirubin'/exp OR bilirubin)            3,031 
#1.  direct AND ('bilirubin'/exp OR bilirubin)               10,555 

Embase provides access to more than 28 million validated biomedical and pharmacological records from Embase and MEDLINE.
This email has been sent to you via Embase, a product of Elsevier Life Sciences IP Limited.

**3. CINAHL**"Biliary atresia AND screening AND ( infant or newborn or neonate)" - 51 results

**4. Cochrane Central**  - Cochrane library**- 111**

1. 'direct bilirubin'
2. 'conjugated bilirubin'
3. ‘fractionated bilirubin’
4. ‘Stool color card’

5. SCC
6. ‘Bile acids’
7. Taurocholate

8. Screening

9. #1 OR #2 OR #3 OR #4 OR #5 OR #6 OR #7 OR #8
10. Explode ‘jaundice’ [all subheadings in MIME, MJME]

11. explode 'biliary atresia' [all subheadings in MIME, MJME]

12. Explode ‘cholestasis’ [all subheadings in MIME, MJME]

13.'Kasai procedure' OR kasai OR ‘portoenterostomy’

14. #10 OR #11 OR #12 OR #13

15. explode 'infant - newborn' [all subheadings in MIME, MJME]
16. neonat*
17. newborn*
18. #15 or #16 or #17

17. #9 AND #14 AND 18
