# Supplementary material for: Population-based screening strategies for biliary atresia in the newborn: A systematic review and meta-analysis
Source: PLoS One. 2024 Aug 28;19(8):e0307837. doi: 10.1371/journal.pone.0307837 (PMC11357077; doi:10.1371/journal.pone.0307837)
Supplement: S2 File — Description of various items extracted from the included studies for the systematic review. (DOCX) [file pone.0307837.s003.docx]

**Supporting Information 2:** Data extraction details

Items extracted-

- Study ID
- Year of pulblication
- Country
- Single/Multicenter
- Study eligibility-Disease
- Study eligibility-Population
- Study eligibility-Test
- Index test
- Threshold
- Assay
- Study design
- Sample description
- Control group?
- Sample size
- Inclusion criteria
- Exclusion criteria
- Blinding?
- True positive
- False positive
- True negative
- False negative
- Area Under the Curve
- Author Contact
- Study conclusion
- Comments (Other)
