# Supplementary material for: Population-based screening strategies for biliary atresia in the newborn: A systematic review and meta-analysis
Source: PLoS One. 2024 Aug 28;19(8):e0307837. doi: 10.1371/journal.pone.0307837 (PMC11357077; doi:10.1371/journal.pone.0307837)
Supplement: S3 File — Detailed description of each study on QUADAS-2 assessment. (DOCX) [file pone.0307837.s004.docx]

**Supporting Information 3: QUADAS-2 assessment of included studies**

1. **Chen 2006:**

**Risk of bias:**

| **Patient selection** | Was a consecutive or random sample of patients enrolled? | Consecutive | Low |
| --- | --- | --- | --- |
|  | Was a case-control design avoided? | Yes |  |
|  | Did the study avoid inappropriate exclusions? | Yes |  |
|  | Could the selection of patients have introduced bias? | No |  |
| **Index test** | Were the index test results interpreted without knowledge of the results of the reference standard? | N/A | Low |
|  | If a threshold was used, was it pre-specified? | Yes |  |
|  | Could the conduct or interpretation of the index test have introduced bias? | No |  |
| **Reference standard** | Is the reference standard likely to correctly classify the target condition? | Yes | Low |
|  | Were the reference standard results interpreted without knowledge of the results of the index test? | N/A |  |
|  | Could the reference standard, its conduct, or its interpretation have introduced bias? | No |  |
| **Flow and timing** | Was there an appropriate interval between index test(s) and reference standard? | N/A | Low |
|  | Did all patients receive a reference standard? | Yes |  |
|  | Did all patients receive the same reference standard? | Yes |  |
|  | Were all patients included in the analysis? | Yes |  |

**Applicability concerns:**

| **Patient selection** | Are there concerns that the included patients do not match the review question? | No | Low |
| --- | --- | --- | --- |
| **Index test** | Are there concerns that the index test, its conduct, or interpretation differ from the review question? | No | Low |
| **Reference standard** | Are there concerns that the target condition as defined by the reference standard does not match the review question? | No | Low |

1. **Gong 2020:**

**Risk of bias:**

| **Patient selection** | Was a consecutive or random sample of patients enrolled? | Consecutive | High |
| --- | --- | --- | --- |
|  | Was a case-control design avoided? | No |  |
|  | Did the study avoid inappropriate exclusions? | Yes |  |
|  | Could the selection of patients have introduced bias? | Yes |  |
| **Index test** | Were the index test results interpreted without knowledge of the results of the reference standard? | No | Unclear |
|  | If a threshold was used, was it pre-specified? | Yes |  |
|  | Could the conduct or interpretation of the index test have introduced bias? | No |  |
| **Reference standard** | Is the reference standard likely to correctly classify the target condition? | Yes | Low |
|  | Were the reference standard results interpreted without knowledge of the results of the index test? | N/A |  |
|  | Could the reference standard, its conduct, or its interpretation have introduced bias? | No |  |
| **Flow and timing** | Was there an appropriate interval between index test(s) and reference standard? | N/A | Unclear |
|  | Did all patients receive a reference standard? | Unclear |  |
|  | Did all patients receive the same reference standard? | Unclear |  |
|  | Were all patients included in the analysis? | Yes |  |

**Applicability concerns:**

| **Patient selection** | Are there concerns that the included patients do not match the review question? | No | Low |
| --- | --- | --- | --- |
| **Index test** | Are there concerns that the index test, its conduct, or interpretation differ from the review question? | No | Low |
| **Reference standard** | Are there concerns that the target condition as defined by the reference standard does not match the review question? | No | Low |

1. **Gu 2015:**

**Risk of bias:**

| **Patient selection** | Was a consecutive or random sample of patients enrolled? | Consecutive | Low |
| --- | --- | --- | --- |
|  | Was a case-control design avoided? | Yes |  |
|  | Did the study avoid inappropriate exclusions? | Yes |  |
|  | Could the selection of patients have introduced bias? | Yes |  |
| **Index test** | Were the index test results interpreted without knowledge of the results of the reference standard? | N/A | Low |
|  | If a threshold was used, was it pre-specified? | Yes |  |
|  | Could the conduct or interpretation of the index test have introduced bias? | No |  |
| **Reference standard** | Is the reference standard likely to correctly classify the target condition? | Yes | Low |
|  | Were the reference standard results interpreted without knowledge of the results of the index test? | N/A |  |
|  | Could the reference standard, its conduct, or its interpretation have introduced bias? | No |  |
| **Flow and timing** | Was there an appropriate interval between index test(s) and reference standard? | N/A | Low |
|  | Did all patients receive a reference standard? | Yes |  |
|  | Did all patients receive the same reference standard? | Yes |  |
|  | Were all patients included in the analysis? | Yes |  |

**Applicability concerns:**

| **Patient selection** | Are there concerns that the included patients do not match the review question? | No | Low |
| --- | --- | --- | --- |
| **Index test** | Are there concerns that the index test, its conduct, or interpretation differ from the review question? | No | Low |
| **Reference standard** | Are there concerns that the target condition as defined by the reference standard does not match the review question? | No | Low |

1. **Gu 2020:**

**Risk of bias:**

| **Patient selection** | Was a consecutive or random sample of patients enrolled? | Consecutive | Low |
| --- | --- | --- | --- |
|  | Was a case-control design avoided? | Yes |  |
|  | Did the study avoid inappropriate exclusions? | Yes |  |
|  | Could the selection of patients have introduced bias? | Yes |  |
| **Index test** | Were the index test results interpreted without knowledge of the results of the reference standard? | N/A | Low |
|  | If a threshold was used, was it pre-specified? | Yes |  |
|  | Could the conduct or interpretation of the index test have introduced bias? | No |  |
| **Reference standard** | Is the reference standard likely to correctly classify the target condition? | Unclear | Unclear |
|  | Were the reference standard results interpreted without knowledge of the results of the index test? | N/A |  |
|  | Could the reference standard, its conduct, or its interpretation have introduced bias? | Unclear |  |
| **Flow and timing** | Was there an appropriate interval between index test(s) and reference standard? | N/A | Unclear |
|  | Did all patients receive a reference standard? | Unclear |  |
|  | Did all patients receive the same reference standard? | Unclear |  |
|  | Were all patients included in the analysis? | Yes |  |

**Applicability concerns:**

| **Patient selection** | Are there concerns that the included patients do not match the review question? | No | Low |
| --- | --- | --- | --- |
| **Index test** | Are there concerns that the index test, its conduct, or interpretation differ from the review question? | No | Low |
| **Reference standard** | Are there concerns that the target condition as defined by the reference standard does not match the review question? | Unclear | Unclear |

1. **Guthery 2019:**

**Risk of bias:**

| **Patient selection** | Was a consecutive or random sample of patients enrolled? | Consecutive | Low |
| --- | --- | --- | --- |
|  | Was a case-control design avoided? | Yes |  |
|  | Did the study avoid inappropriate exclusions? | Yes |  |
|  | Could the selection of patients have introduced bias? | Yes |  |
| **Index test** | Were the index test results interpreted without knowledge of the results of the reference standard? | N/A | Low |
|  | If a threshold was used, was it pre-specified? | Yes |  |
|  | Could the conduct or interpretation of the index test have introduced bias? | No |  |
| **Reference standard** | Is the reference standard likely to correctly classify the target condition? | Unclear | Unclear |
|  | Were the reference standard results interpreted without knowledge of the results of the index test? | N/A |  |
|  | Could the reference standard, its conduct, or its interpretation have introduced bias? | Unclear |  |
| **Flow and timing** | Was there an appropriate interval between index test(s) and reference standard? | N/A | Unclear |
|  | Did all patients receive a reference standard? | Unclear |  |
|  | Did all patients receive the same reference standard? | Unclear |  |
|  | Were all patients included in the analysis? | Yes |  |

**Applicability concerns:**

| **Patient selection** | Are there concerns that the included patients do not match the review question? | No | Low |
| --- | --- | --- | --- |
| **Index test** | Are there concerns that the index test, its conduct, or interpretation differ from the review question? | No | Low |
| **Reference standard** | Are there concerns that the target condition as defined by the reference standard does not match the review question? | Unclear | Unclear |

1. **Harpavat 2016:**

**Risk of bias:**

| **Patient selection** | Was a consecutive or random sample of patients enrolled? | Consecutive | Low |
| --- | --- | --- | --- |
|  | Was a case-control design avoided? | Yes |  |
|  | Did the study avoid inappropriate exclusions? | Yes |  |
|  | Could the selection of patients have introduced bias? | Yes |  |
| **Index test** | Were the index test results interpreted without knowledge of the results of the reference standard? | N/A | Low |
|  | If a threshold was used, was it pre-specified? | Yes |  |
|  | Could the conduct or interpretation of the index test have introduced bias? | No |  |
| **Reference standard** | Is the reference standard likely to correctly classify the target condition? | Yes | Low |
|  | Were the reference standard results interpreted without knowledge of the results of the index test? | N/A |  |
|  | Could the reference standard, its conduct, or its interpretation have introduced bias? | No |  |
| **Flow and timing** | Was there an appropriate interval between index test(s) and reference standard? | N/A | Low |
|  | Did all patients receive a reference standard? | Yes |  |
|  | Did all patients receive the same reference standard? | Yes |  |
|  | Were all patients included in the analysis? | Yes |  |

**Applicability concerns:**

| **Patient selection** | Are there concerns that the included patients do not match the review question? | No | Low |
| --- | --- | --- | --- |
| **Index test** | Are there concerns that the index test, its conduct, or interpretation differ from the review question? | No | Low |
| **Reference standard** | Are there concerns that the target condition as defined by the reference standard does not match the review question? | No | Low |

1. **Harpavat 2020:**

**Risk of bias:**

| **Patient selection** | Was a consecutive or random sample of patients enrolled? | Consecutive | Low |
| --- | --- | --- | --- |
|  | Was a case-control design avoided? | Yes |  |
|  | Did the study avoid inappropriate exclusions? | Yes |  |
|  | Could the selection of patients have introduced bias? | Yes |  |
| **Index test** | Were the index test results interpreted without knowledge of the results of the reference standard? | N/A | Low |
|  | If a threshold was used, was it pre-specified? | Yes |  |
|  | Could the conduct or interpretation of the index test have introduced bias? | No |  |
| **Reference standard** | Is the reference standard likely to correctly classify the target condition? | Yes | Low |
|  | Were the reference standard results interpreted without knowledge of the results of the index test? | N/A |  |
|  | Could the reference standard, its conduct, or its interpretation have introduced bias? | No |  |
| **Flow and timing** | Was there an appropriate interval between index test(s) and reference standard? | N/A | Low |
|  | Did all patients receive a reference standard? | Yes |  |
|  | Did all patients receive the same reference standard? | Yes |  |
|  | Were all patients included in the analysis? | Yes |  |

**Applicability concerns:**

| **Patient selection** | Are there concerns that the included patients do not match the review question? | No | Low |
| --- | --- | --- | --- |
| **Index test** | Are there concerns that the index test, its conduct, or interpretation differ from the review question? | No | Low |
| **Reference standard** | Are there concerns that the target condition as defined by the reference standard does not match the review question? | No | Low |

1. **Hsiao 2008:**

**Risk of bias:**

| **Patient selection** | Was a consecutive or random sample of patients enrolled? | Consecutive | Low |
| --- | --- | --- | --- |
|  | Was a case-control design avoided? | Yes |  |
|  | Did the study avoid inappropriate exclusions? | Yes |  |
|  | Could the selection of patients have introduced bias? | Yes |  |
| **Index test** | Were the index test results interpreted without knowledge of the results of the reference standard? | N/A | Low |
|  | If a threshold was used, was it pre-specified? | Yes |  |
|  | Could the conduct or interpretation of the index test have introduced bias? | No |  |
| **Reference standard** | Is the reference standard likely to correctly classify the target condition? | Yes | Low |
|  | Were the reference standard results interpreted without knowledge of the results of the index test? | N/A |  |
|  | Could the reference standard, its conduct, or its interpretation have introduced bias? | No |  |
| **Flow and timing** | Was there an appropriate interval between index test(s) and reference standard? | N/A | Low |
|  | Did all patients receive a reference standard? | Yes |  |
|  | Did all patients receive the same reference standard? | Yes |  |
|  | Were all patients included in the analysis? | Yes |  |

**Applicability concerns:**

| **Patient selection** | Are there concerns that the included patients do not match the review question? | No | Low |
| --- | --- | --- | --- |
| **Index test** | Are there concerns that the index test, its conduct, or interpretation differ from the review question? | No | Low |
| **Reference standard** | Are there concerns that the target condition as defined by the reference standard does not match the review question? | No | Low |

1. **Kastenburg 2023:**

**Risk of bias:**

| **Patient selection** | Was a consecutive or random sample of patients enrolled? | Consecutive | Low |
| --- | --- | --- | --- |
|  | Was a case-control design avoided? | Yes |  |
|  | Did the study avoid inappropriate exclusions? | Yes |  |
|  | Could the selection of patients have introduced bias? | Yes |  |
| **Index test** | Were the index test results interpreted without knowledge of the results of the reference standard? | N/A | Low |
|  | If a threshold was used, was it pre-specified? | Yes |  |
|  | Could the conduct or interpretation of the index test have introduced bias? | No |  |
| **Reference standard** | Is the reference standard likely to correctly classify the target condition? | Yes | Low |
|  | Were the reference standard results interpreted without knowledge of the results of the index test? | N/A |  |
|  | Could the reference standard, its conduct, or its interpretation have introduced bias? | No |  |
| **Flow and timing** | Was there an appropriate interval between index test(s) and reference standard? | N/A | Low |
|  | Did all patients receive a reference standard? | Yes |  |
|  | Did all patients receive the same reference standard? | Yes |  |
|  | Were all patients included in the analysis? | Yes |  |

**Applicability concerns:**

| **Patient selection** | Are there concerns that the included patients do not match the review question? | No | Low |
| --- | --- | --- | --- |
| **Index test** | Are there concerns that the index test, its conduct, or interpretation differ from the review question? | No | Low |
| **Reference standard** | Are there concerns that the target condition as defined by the reference standard does not match the review question? | No | Low |

1. **Kong 2016:**

**Risk of bias:**

| **Patient selection** | Was a consecutive or random sample of patients enrolled? | Consecutive | Low |
| --- | --- | --- | --- |
|  | Was a case-control design avoided? | Yes |  |
|  | Did the study avoid inappropriate exclusions? | Yes |  |
|  | Could the selection of patients have introduced bias? | Yes |  |
| **Index test** | Were the index test results interpreted without knowledge of the results of the reference standard? | N/A | Low |
|  | If a threshold was used, was it pre-specified? | Yes |  |
|  | Could the conduct or interpretation of the index test have introduced bias? | No |  |
| **Reference standard** | Is the reference standard likely to correctly classify the target condition? | Yes | Low |
|  | Were the reference standard results interpreted without knowledge of the results of the index test? | N/A |  |
|  | Could the reference standard, its conduct, or its interpretation have introduced bias? | No |  |
| **Flow and timing** | Was there an appropriate interval between index test(s) and reference standard? | N/A | Low |
|  | Did all patients receive a reference standard? | Yes |  |
|  | Did all patients receive the same reference standard? | Yes |  |
|  | Were all patients included in the analysis? | Yes |  |

**Applicability concerns:**

| **Patient selection** | Are there concerns that the included patients do not match the review question? | No | Low |
| --- | --- | --- | --- |
| **Index test** | Are there concerns that the index test, its conduct, or interpretation differ from the review question? | No | Low |
| **Reference standard** | Are there concerns that the target condition as defined by the reference standard does not match the review question? | No | Low |

1. **Matsui 1993:**

**Risk of bias:**

| **Patient selection** | Was a consecutive or random sample of patients enrolled? | Consecutive | Low |
| --- | --- | --- | --- |
|  | Was a case-control design avoided? | Yes |  |
|  | Did the study avoid inappropriate exclusions? | Yes |  |
|  | Could the selection of patients have introduced bias? | Yes |  |
| **Index test** | Were the index test results interpreted without knowledge of the results of the reference standard? | N/A | Low |
|  | If a threshold was used, was it pre-specified? | Yes |  |
|  | Could the conduct or interpretation of the index test have introduced bias? | No |  |
| **Reference standard** | Is the reference standard likely to correctly classify the target condition? | Yes | Low |
|  | Were the reference standard results interpreted without knowledge of the results of the index test? | N/A |  |
|  | Could the reference standard, its conduct, or its interpretation have introduced bias? | No |  |
| **Flow and timing** | Was there an appropriate interval between index test(s) and reference standard? | N/A | Low |
|  | Did all patients receive a reference standard? | Yes |  |
|  | Did all patients receive the same reference standard? | Yes |  |
|  | Were all patients included in the analysis? | Yes |  |

**Applicability concerns:**

| **Patient selection** | Are there concerns that the included patients do not match the review question? | No | Low |
| --- | --- | --- | --- |
| **Index test** | Are there concerns that the index test, its conduct, or interpretation differ from the review question? | No | Low |
| **Reference standard** | Are there concerns that the target condition as defined by the reference standard does not match the review question? | No | Low |

1. **Powell 2003:**

**Risk of bias:**

| **Patient selection** | Was a consecutive or random sample of patients enrolled? | Consecutive | Low |
| --- | --- | --- | --- |
|  | Was a case-control design avoided? | Yes |  |
|  | Did the study avoid inappropriate exclusions? | Yes |  |
|  | Could the selection of patients have introduced bias? | Yes |  |
| **Index test** | Were the index test results interpreted without knowledge of the results of the reference standard? | N/A | Low |
|  | If a threshold was used, was it pre-specified? | Yes |  |
|  | Could the conduct or interpretation of the index test have introduced bias? | No |  |
| **Reference standard** | Is the reference standard likely to correctly classify the target condition? | Unclear | Unclear |
|  | Were the reference standard results interpreted without knowledge of the results of the index test? | N/A |  |
|  | Could the reference standard, its conduct, or its interpretation have introduced bias? | Unclear |  |
| **Flow and timing** | Was there an appropriate interval between index test(s) and reference standard? | N/A | Unclear |
|  | Did all patients receive a reference standard? | Unclear |  |
|  | Did all patients receive the same reference standard? | Unclear |  |
|  | Were all patients included in the analysis? | Yes |  |

**Applicability concerns:**

| **Patient selection** | Are there concerns that the included patients do not match the review question? | No | Low |
| --- | --- | --- | --- |
| **Index test** | Are there concerns that the index test, its conduct, or interpretation differ from the review question? | No | Low |
| **Reference standard** | Are there concerns that the target condition as defined by the reference standard does not match the review question? | Unclear | Unclear |

1. **Schreiber 2014:**

**Risk of bias:**

| **Patient selection** | Was a consecutive or random sample of patients enrolled? | Consecutive | Low |
| --- | --- | --- | --- |
|  | Was a case-control design avoided? | Yes |  |
|  | Did the study avoid inappropriate exclusions? | Yes |  |
|  | Could the selection of patients have introduced bias? | Yes |  |
| **Index test** | Were the index test results interpreted without knowledge of the results of the reference standard? | N/A | Low |
|  | If a threshold was used, was it pre-specified? | Yes |  |
|  | Could the conduct or interpretation of the index test have introduced bias? | No |  |
| **Reference standard** | Is the reference standard likely to correctly classify the target condition? | Unclear | Unclear |
|  | Were the reference standard results interpreted without knowledge of the results of the index test? | N/A |  |
|  | Could the reference standard, its conduct, or its interpretation have introduced bias? | Unclear |  |
| **Flow and timing** | Was there an appropriate interval between index test(s) and reference standard? | N/A | Unclear |
|  | Did all patients receive a reference standard? | Unclear |  |
|  | Did all patients receive the same reference standard? | Unclear |  |
|  | Were all patients included in the analysis? | Yes |  |

**Applicability concerns:**

| **Patient selection** | Are there concerns that the included patients do not match the review question? | No | Low |
| --- | --- | --- | --- |
| **Index test** | Are there concerns that the index test, its conduct, or interpretation differ from the review question? | No | Low |
| **Reference standard** | Are there concerns that the target condition as defined by the reference standard does not match the review question? | No | Unclear |

1. **Suzuki 2011:**

**Risk of bias:**

| **Patient selection** | Was a consecutive or random sample of patients enrolled? | Consecutive | Low |
| --- | --- | --- | --- |
|  | Was a case-control design avoided? | Yes |  |
|  | Did the study avoid inappropriate exclusions? | Yes |  |
|  | Could the selection of patients have introduced bias? | Yes |  |
| **Index test** | Were the index test results interpreted without knowledge of the results of the reference standard? | N/A | Low |
|  | If a threshold was used, was it pre-specified? | Yes |  |
|  | Could the conduct or interpretation of the index test have introduced bias? | No |  |
| **Reference standard** | Is the reference standard likely to correctly classify the target condition? | Yes | Low |
|  | Were the reference standard results interpreted without knowledge of the results of the index test? | N/A |  |
|  | Could the reference standard, its conduct, or its interpretation have introduced bias? | No |  |
| **Flow and timing** | Was there an appropriate interval between index test(s) and reference standard? | N/A | Low |
|  | Did all patients receive a reference standard? | Yes |  |
|  | Did all patients receive the same reference standard? | Yes |  |
|  | Were all patients included in the analysis? | Yes |  |

**Applicability concerns:**

| **Patient selection** | Are there concerns that the included patients do not match the review question? | No | Low |
| --- | --- | --- | --- |
| **Index test** | Are there concerns that the index test, its conduct, or interpretation differ from the review question? | No | Low |
| **Reference standard** | Are there concerns that the target condition as defined by the reference standard does not match the review question? | No | Low |

1. **Woolfson 2018:**

**Risk of bias:**

| **Patient selection** | Was a consecutive or random sample of patients enrolled? | Consecutive | Low |
| --- | --- | --- | --- |
|  | Was a case-control design avoided? | Yes |  |
|  | Did the study avoid inappropriate exclusions? | Yes |  |
|  | Could the selection of patients have introduced bias? | Yes |  |
| **Index test** | Were the index test results interpreted without knowledge of the results of the reference standard? | N/A | Low |
|  | If a threshold was used, was it pre-specified? | Yes |  |
|  | Could the conduct or interpretation of the index test have introduced bias? | No |  |
| **Reference standard** | Is the reference standard likely to correctly classify the target condition? | Yes | Low |
|  | Were the reference standard results interpreted without knowledge of the results of the index test? | N/A |  |
|  | Could the reference standard, its conduct, or its interpretation have introduced bias? | No |  |
| **Flow and timing** | Was there an appropriate interval between index test(s) and reference standard? | N/A | Low |
|  | Did all patients receive a reference standard? | Yes |  |
|  | Did all patients receive the same reference standard? | Yes |  |
|  | Were all patients included in the analysis? | Yes |  |

**Applicability concerns:**

| **Patient selection** | Are there concerns that the included patients do not match the review question? | No | Low |
| --- | --- | --- | --- |
| **Index test** | Are there concerns that the index test, its conduct, or interpretation differ from the review question? | No | Low |
| **Reference standard** | Are there concerns that the target condition as defined by the reference standard does not match the review question? | No | Low |
