# Supplementary material for: Population-based screening strategies for biliary atresia in the newborn: A systematic review and meta-analysis
Source: PLoS One. 2024 Aug 28;19(8):e0307837. doi: 10.1371/journal.pone.0307837 (PMC11357077; doi:10.1371/journal.pone.0307837)
Supplement: S4 File — Details on studies that were excluded from the metanalysis and reasons for exclusion. (DOCX) [file pone.0307837.s005.docx]

**Supporting Information 4**: Characteristics of excluded studies

|  | **Study** | **Year** | **Index Test** | **Reason for Exclusion** |
| --- | --- | --- | --- | --- |
|  | Akiyama et al (1) | 1994 | NIRS on fecal samples | Not population based |
|  | Chang et al (2) | 2006 | SCC | Not population based |
|  | Chiu et al (3) | 2013 | SCC | Not population based |
|  | Dai et al (4) | 2021 | USG Nomogram | Not population based |
|  | El-Shabrawi et al (5) | 2021 | SCC | Not population based |
|  | Gaio et al (6) | 2016 | SCC | Not population based |
|  | Gu et al (7) | 2017 | SCC | Not population based |
|  | Gustafson et al (8) | 2006 | Bile Acid | Not population based |
|  | Harpavat et al (9) | 2011 | DB/CB | Conference abstract of paper included |
|  | Harpavat et al(10) | 2011 | DB/CB | Not population based |
|  | Harpavat et al(11) | 2016 | DB/CB | Not population based |
|  | Harpavat et al(12) | 2018 | DB/CB | Not population based |
|  | Hoshino et al(13) | 2017 | RBG Hue saturation on iPhone | Not population-based |
|  | Ikegawa et al(14) | 1994 | Conjugated CA-1 beta-ol via RIA | Not population-based |
|  | Lee et al(15) | 2016 | SCC | Not population-based |
|  | Liao et al(16) | 2022 | DB | Not population based |
|  | Lein et al(17) | 2011 | SCC | Not population-based |
|  | Matsui et al(18) | 1996 | USBA | Not population-based |
|  | Morinville et al(19) | 2016 | SCC | Not population based |
|  | Noorulla et al(20) | 2019 | Direct Bilirubin | Research letter/case series/retrospective study |
|  | Rabbani et al(21) | 2021 | Physical exam/SCC/Fractionated bilirubin | Review article |
|  | Ramonet et al(22) | 2013 | SCC | Not screening for primary disease (BA) |
|  | Sasaki et al(23) | 1984 | CGA and CDCA dried blood via RIA | Not population-based |
|  | Shneider et al (24) | 2017 | DB/CB | Not population-based |
|  | Scrieber et al (25) | 2012 | Home based screening of SCC | Conference abstract (Main paper included) |
|  | Shen et al (26) | 2016 | Stool color app | Not population-based |
|  | Shen et al (27) | 2020 | GGT+ Liver stiffness measurement | Not population based |
|  | Small et al (28) | 2018 | Retrospective study on cholestasis | Not screening for primary disease (BA) |
|  | Tseng et al (29) | 2011 | SCC | Not population based |
|  | Watayo et al (30) | 2013 | SCC | Clinical trial ongoing |
|  | Woolfson et al (31) | 2017 | SCC | Conference abstract (Main paper included) |
|  | Xiao et al (32) | 2020 | Targeted metabolites | Not population based |
|  | Zhao et al (33) | 2020 | Bile acid scoring system | Not population based |
|  | Zhao et al (34) | 2021 | Web based calculation using clinical, lab and USG findings | Not population based |
|  | Zheng et al(35) | 2020 | SCC | Not population based |
|  | Zhou et al (36) | 2012 | Bile acids | Not population based |

**References to excluded studies**

1. Akiyama T, Yamauchi Y. Use of near infrared reflectance spectroscopy in the screening for biliary atresia. Journal of pediatric surgery. 1994 May 1;29(5):645-7.
2. Chang M. Screening for biliary atresia. Chang Gung medical journal. 2006 May 1;29(3):231.
3. Chiu CY, Chen PH, Chan CF, Chang MH, Wu TC, Taiwan Infant Stool Color Card Study Group. Biliary atresia in preterm infants in Taiwan: a nationwide survey. The Journal of pediatrics. 2013 Jul 1;163(1):100-3.
4. Dai SY, Sun YQ, Wu Y, Chen G, Sun S, Dong R, Zheng S. Development and assessment of screening nomogram for biliary atresia based on hepatobiliary ultrasonographic features. Frontiers in Pediatrics. 2021 May 17;9:625451.
5. El-Shabrawi MH, Baroudy SR, Hassanin FS, Farag AE. A pilot study of the value of a stool color card as a diagnostic tool for extrahepatic biliary atresia at a single tertiary referral center in a low/middle income country. Arab Journal of Gastroenterology. 2021 Mar 1;22(1):61-5.
6. Gaio P, Pergola E, Rosellini E, Boscardin C, Gamba P, Cananzi M. Stool colour cards allow specific identification of infant cholestasis: results of a prospective, experimental, monocentric, non-profit pilot study [abstract]. In: ESPGHAN 49^th^ Annual Meeting; May 2016; Athens, Greece.
7. Gu YH, Matsui A. Long‐term native liver survival in infants with biliary atresia and use of a stool color card: Case–control study. Pediatrics International. 2017 Nov;59(11):1189-93.
8. Gustafsson J, Alvelius G, Björkhem I, Nemeth A. Bile Acid Metabolism in Extrahepatic Biliary Atresia. Upsala journal of medical sciences. 2006 Jan 1;111(1):131-6.
9. Harpavat S, Finegold MJ, Karpen SJ. Biliary Atresia Patients have elevated direct/conjugated bilirubin levels shortly after birth. (2011), Abstracts. Hepatology, 54: 360A-1455A. <https://doi.org/10.1002/hep.24666>
10. Harpavat S, Finegold MJ, Karpen SJ. Patients with biliary atresia have elevated direct/conjugated bilirubin levels shortly after birth. Pediatrics. 2011 Dec;128(6):e1428-33.
11. Harpavat S, Ramraj R, Finegold MJ, Brandt ML, Hertel PM, Fallon SC, et al. Newborn direct or conjugated bilirubin measurements as a potential screen for biliary atresia. J Pediatr Gastroenterol Nutr. 2016 Jun 1;62(6):799–803.
12. Harpavat S, Lupo PJ, Liwanag L, Hollier J, Brandt ML, Finegold MJ, et al. Factors Influencing Time-to-diagnosis of Biliary Atresia. J Pediatr Gastroenterol Nutr. 2018 Jun 1;66(6):850–6.
13. Hoshino E, Hayashi K, Suzuki M, Obatake M, Urayama KY, Nakano S, et al. An iPhone application using a novel stool color detection algorithm for biliary atresia screening. Pediatr Surg Int. 2017 Oct 1;33(10):1115–21.
14. IKEGAWA S, MURAI T, MATSUI A, TOHMA M. Radioimmunoassay of conjugated 1β-hydroxycholic acid in dried blood spots for diagnosis of congenital biliary atresia. Biological and Pharmaceutical Bulletin. 1994 Jan 15;17(1):5-8.
15. Lee M, Chen SCC, Yang HY, Huang JH, Yeung CY, Lee HC. Infant stool color card screening helps reduce the hospitalization rate and mortality of biliary atresia a 14-year nationwide cohort study in Taiwan. Medicine (United States). 2016;95(12).
16. Liao FM, Chang KC, Wu JF, Chen HL, Ni YH, Chang MH. Direct Bilirubin and Risk of Biliary Atresia. Pediatrics. 2022 Jun 1;149(6).
17. Lien TH, Chang MH, Wu JF, Chen HL, Lee HC, Chen AC, et al. Effects of the infant stool color card screening program on 5-year outcome of biliary Atresia in Taiwan. Hepatology. 2011 Jan;53(1):202–8.
18. Matsui A, Kasano Y, Yamauchi Y, Momoya T, Shimada T, Ishikawa T, Abukawa D, Kimura A, Adachi K, Tazuke Y. Direct enzymatic assay of urinary sulfated bile acids to replace serum bilirubin testing for selective screening of neonatal cholestasis. The Journal of pediatrics. 1996 Aug 1;129(2):306-8.
19. Morinville V, Ahmed N, Ibberson C, Kovacs L, Kaczorowski J, Bryan S, et al. Home-based screening for biliary atresia using infant stool color cards in Canada: Quebec feasibility study. J Pediatr Gastroenterol Nutr. 2016;62(4):536–41.
20. Noorulla F, Dedon R, Maisels MJ. Association of Early Direct Bilirubin Levels and Biliary Atresia among Neonates. JAMA Netw Open. 2019 Oct 16;2(10).
21. Rabbani T, Guthery SL, Himes R, Shneider BL, Harpavat S. Newborn screening for biliary atresia: A review of current methods. Current Gastroenterology Reports. 2021 Dec;23:1-8.
22. Ramonet MD, Gómez S, Morise S, Parga L, Caglio P, De Micheli M, et al. Detección precoz de la colestasis neonatal en las heces por el método de tamizaje con tarjetas colorimétricas. Arch Argent Pediatr. 2013 Apr;111(2):135–8.
23. Sasaki H. Development of Bile Acid Metabolism in Neonates during Perinatal Period| Part 2. Mass screening of congenital biliary atresia by radioimmunoassay using dried blood spot. Pediatrics International. 1984 Jun;26(2):161-8.
24. Shneider BL, Moore J, Kerkar N, Magee JC, Ye W, Karpen SJ, et al. Initial assessment of the infant with neonatal cholestasis-Is this biliary atresia? PLoS One. 2017 May 1;12(5).
25. Schreiber RA, Kaczorowski J, Lutley P, Bryan S, Collet JP. Home-Based Screening for Biliary Atresia using Infant Stool Color Cards: A Large Scale Feasibility Study (2012), AASLD Abstracts. Hepatology, 56: 191A-1144A. <https://doi.org/10.1002/hep.26040>
26. Shen Z, Zheng S, Dong R, Chen G. Saturation of stool color in HSV color model is a promising objective parameter for screening biliary atresia. J Pediatr Surg. 2016 Dec 1;51(12):2091–4.
27. Shen Q, Tan SS, Wang Z, Cai S, Pang W, Peng C, et al. Combination of gamma-glutamyl transferase and liver stiffness measurement for biliary atresia screening at different ages: A retrospective analysis of 282 infants. BMC Pediatr. 2020 Jun 4;20(1).
28. Small et al. (2018), Posters (Abstracts 301–2389). Hepatology, 68: 184-1353. <https://doi.org/10.1002/hep.30257>
29. Tseng JJ, Lai MS, Lin MC, Fu YC. Stool color card screening for biliary atresia. Pediatrics. 2011 Nov;128(5).
30. Watayo H. Screening for biliary atresia using digital photos with stool color card. 2013. <https://rctportal.niph.go.jp/en/detail?trial_id=UMIN000011224>
31. Woolfson J, Schreiber R, Butler A, MacFarlane J, Kaczorowski J, Masucci L, Bryan S, Collet JP. Biliary Atresia Home Screening Program In British Columbia: Evaluation Of First Two Years. 94th Annual Conference of the Canadian Paediatric Society, 2017
32. Xiao Y, Zhou Y, Zhou K, Cai W. Targeted Metabolomics Reveals Birth Screening Biomarkers for Biliary Atresia in Dried Blood Spots. J Proteome Res. 2022 Mar 4;21(3):721–6.
33. Zhao D, Zhou K, Chen Y, Xie W, Zhang Y. Development and validation of bile acid profile-based scoring system for identification of biliary atresia: A prospective study. BMC Pediatr. 2020 May 27;20(1).
34. Zhao D, Gu S, Gong X, Li Y, Sun X, Chen Y, et al. Web-based calculator for biliary atresia screening in neonates and infants with cholestasis. Transl Pediatr. 2021 Feb 1;10(2):225–35.
35. Zheng J, Ye Y, Wang B, Zhang L. Biliary atresia screening in Shenzhen: Implementation and achievements. Arch Dis Child. 2020 Aug 1;105(8):720–3.
36. Zhou K, Lin N, Xiao Y, Wang Y, Wen J, Zou GM, et al. Elevated Bile Acids in Newborns with Biliary Atresia (BA). PLoS One. 2012 Nov 14;7(11).
