# Supplementary figures and images for: Population-based screening strategies for biliary atresia in the newborn: A systematic review and meta-analysis
Source: PLoS One. 2024 Aug 28;19(8):e0307837. doi: 10.1371/journal.pone.0307837 (PMC11357077; doi:10.1371/journal.pone.0307837)

**Supporting Information 6: GRADE certainty of evidence rating for DB/CB and SCC screening**


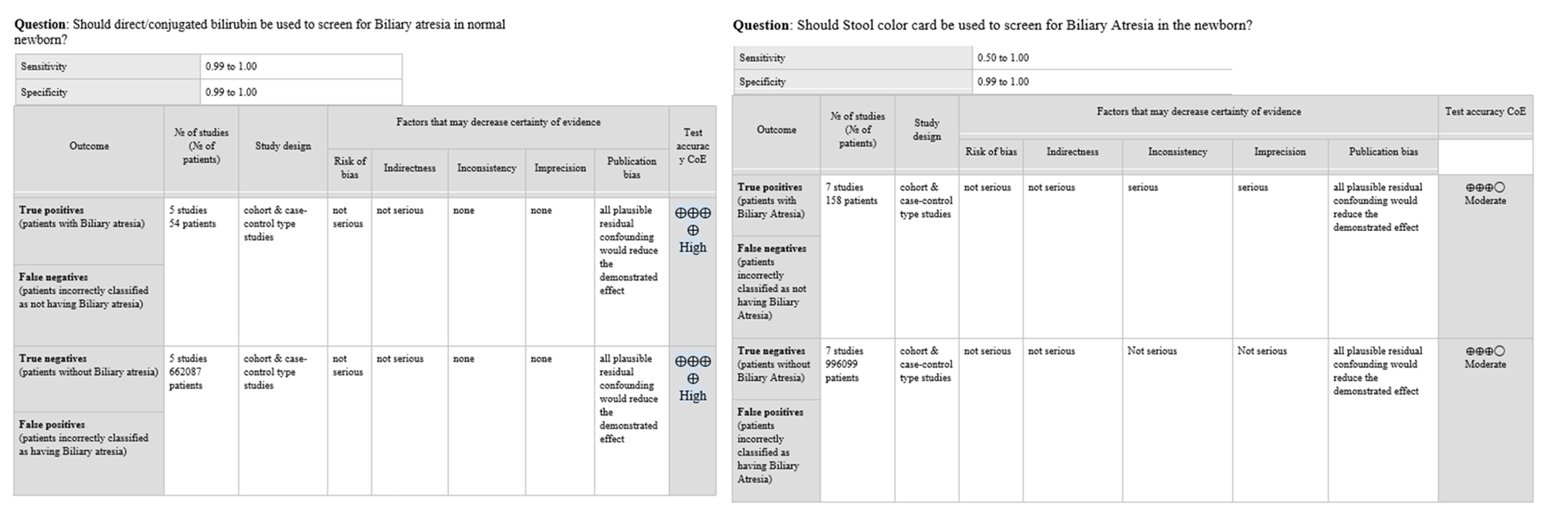

Supplement: S5 File — Tables showing GRADE certainty of evidence for both direct/conjugated bilirubin and Stool Color Card test for screening of biliary atresia. (DOCX) [file pone.0307837.s006.docx]
