# Supplementary material for: Population-based screening strategies for biliary atresia in the newborn: A systematic review and meta-analysis
Source: PLoS One. 2024 Aug 28;19(8):e0307837. doi: 10.1371/journal.pone.0307837 (PMC11357077; doi:10.1371/journal.pone.0307837)
Supplement: S1 Table — (DOCX) [file pone.0307837.s007.docx]

**Supplementary Information 5**: Table. Summary sensitivities and specificities of screening strategies for biliary atresia.

| **Test** | **Studies** | **n/N** | **Sensitivity (95% CI)** | **Specificity (95% CI)** |
| --- | --- | --- | --- | --- |
| SCC | 7 | 165/996262 | 79.6 (70.6, 86.4) | 99.9 (99.9, 99.9) |
| DB/CB | 5 | 54/662141 | 100.0^a^ (100.0, 100.0) | 98.8^a^ (98.8, 98.9) |

Sensitivities and specificities are expressed as percentages.

^a^Estimates and confidence intervals calculated using the sum of the number of true positives, false positives, false negatives and true negatives across the 2x2 tables.
